# Nephropathogenic Infectious Bronchitis Virus Mediates Kidney Injury in Chickens *via* the TLR7/NF-κB Signaling Axis

**DOI:** 10.3389/fcimb.2022.865283

**Published:** 2022-03-23

**Authors:** Ning Li, Cheng Huang, Wei Chen, Zhengqing Li, Guoliang Hu, Guyue Li, Ping Liu, Ruiming Hu, Yu Zhuang, Junrong Luo, Xiaona Gao, Xiaoquan Guo

**Affiliations:** Jiangxi Provincial Key Laboratory for Animal Health, Institute of Animal Population Health, College of Animal Science and Technology, Jiangxi Agricultural University, Nanchang, China

**Keywords:** chicken, nephropathogenic infectious bronchitis virus, TLR7/NF-κB signaling axis, innate immunity, inflammation, kidney injury

## Abstract

Nephropathogenic infectious bronchitis virus (NIBV) is one of the most important viral pathogens in the world poultry industry. Here, we used RT–qPCR, WB and immunofluorescence to explore the interaction between NIBV and the host innate immune system of the kidney. Multiple virions were found in the kidney tissues of the disease group under electron microscopy, and pathological changes such as structural damage of renal tubules and bleeding were observed by HE staining. In addition, we found that the mRNA levels of TLR7, TRAF6, and IKKβ were upregulated after NIBV infection. IRF7 mRNA levels decreased significantly at 5 dpi and increased significantly at 11 to 18 dpi. The NF-κB P65 mRNA level increased significantly at 5 to 18 dpi and decreased at 28 dpi. However, NIBV infection-induced NF-κB P65 protein levels were downregulated at multiple time points. Moreover, we demonstrated that the cytokine (IFN-γ, IL-8, and IL-6) mRNA and protein expression levels were increased significantly at multiple time points after NIBV infection. Furthermore, immunofluorescence analysis showed that NF-κB P65 and IFN-γ were mainly located in the nuclear or perinuclear region. The positive signal intensity of NF-κB P65 was significantly lower than that of the normal group at 1 to 5 dpi, and there was no significant change in the subsequent time period. The positive signal intensity of IFN-γ decreased significantly at 5 dpi, and increased significantly at 11 to 28 dpi. In conclusion, we found that NIBV promoted cytokine release through the TLR7/NF-κB signaling axis, thus causing kidney injury.

## Introduction

Nephropathogenic infectious bronchitis virus (NIBV) has become the most common IBV strain in the commercial poultry industry, which is highly infectious and spreads quickly (He et al., 2016a; Kuang et al., 2020). The genome of this virus is a single plus-stranded RNA that belongs to the genus Gammacoronavirus, family Coronaviridae, in the order Nidovirales (Bayry et al., 2005). The NIBV strain was first reported in the United States and Australia (Winterfield and Hitchner, 1962; Song et al., 1998), and it is now being reported in other parts of the world, especially in Asian and Middle Eastern countries (Lim et al., 2011; Bande et al., 2017; Jiang et al., 2020). All ages of chickens can be infected, and infected chickens are characterized by coughing, sneezing, decreased egg production and intestinal symptoms; at autopsy, the kidneys are pale and enlarged. The case fatality rate can reach 5%–30%, resulting in serious economic losses for the poultry breeding industry (Bing et al., 2007; Li et al., 2020).

Chicken kidneys are the main target organ of NIBV. A large number of studies have shown that NIBV replicates in renal tubular epithelial cells and can cause renal structural changes (Han et al., 2011; Huang et al., 2017; Zhu et al., 2020). Our previous study proved that NIBV infection could lead to decreased antioxidant capacity and metabolic dysfunction in the kidney (Xu et al., 2019). Transcriptomic analysis of the kidneys of IBV-infected animals revealed local activation of several innate immune genes, including Toll receptors, cytokines and chemokines (Smith et al., 2015; He et al., 2016b). However, the relationship between NIBV and the innate immune system of the host remains unclear, as does the specific mechanism of the innate immune response to NIBV infection through the TLR7/NF-κB signaling axis.

Toll-like receptor 7 (TLR7) is localized to the endosomal compartment, where it binds to microorganisms or to self-derived single-stranded RNA (ssRNA) ligands (Petes et al., 2017). It is well known that viruses stimulate Toll receptors, leading to the nuclear translocation of nuclear factors and the activation of inflammatory cytokines and chemokines such as IL-6 and IL-8 (Li et al., 2013; Gimeno et al., 2021). Recently, it has been demonstrated that the type II IFN signaling pathway is indispensable in TLR7 for promoting autoreactive B cell development and systemic immunity (Chodisetti et al., 2020). Moreover, multiple studies have indicated that IFN-γ is critical in the development of coronaviral hepatitis (Kim et al., 2007) and is one of the major cytokines elevated in SARS patients (Yang et al., 2014). In addition, the TLR7/NF-κB signaling axis was shown to be closely related to nephritis, renal insufficiency and nephrotic syndrome (Lin et al., 2017; Zhu et al., 2020; Abaidullah et al., 2021). However, the role of the TLR7/NF-κB signaling axis in the kidney tissues of chickens infected with NIBV remains unclear.

In the present study, we established a model of NIBV infection to investigate the role of NIBV infection on the TLR7/NF-κB signaling axis of the kidney. Our findings indicated that NIBV infection induced TLR7/NF-κB signaling axis activation and promoted the expression of cytokines in the kidney. This study addresses TLR7/NF-κB signaling pathway responses to NIBV in chickens and provides essential information to further improve our understanding of the immune pathogenic mechanism of avian coronaviruses.

## Materials and Methods

### Virus Strain

The virulent IBV strain used was the *SX9* strain, which was isolated and preserved from the College of Animal Science and Technology, Jiangxi Agriculture University.

### Experimental Design

**Figure 1:**
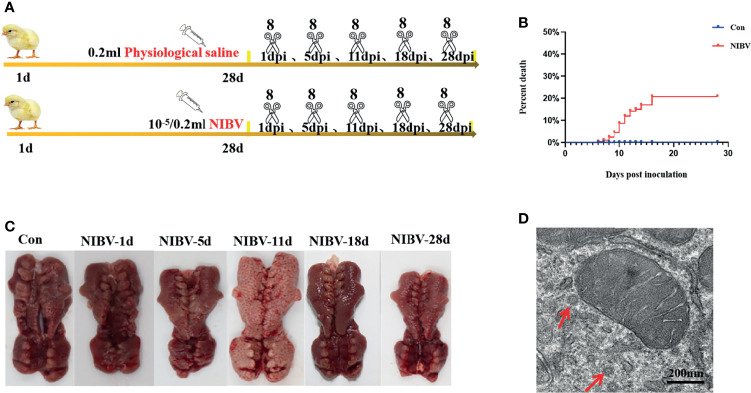
Changes of kidney in chickens infected with nephropathogenic infectious bronchitis virus (NIBV) SX9 strain (10^-5^/0.2 ml). **(A)** Experimental design. Including electron microscope and pathological section were observed, as well as RT-qPCR, western blot and confocal immunofluorescence analysis; **(B)** Mortality rate of 1 dpi-28 dpi chickens infected with NIBV; **(C)** Renal necropsy observation; **(D)** Ultrastructural of renal tissue, the red arrow points to the virus particle.

A total of 300 one-day-old Hy-Line brown laying hens were randomly divided into two groups—the control group (Con, 100) and the disease group (Dis, 200)—which were fed *ad libitum* with diet (all the nutrients in the feed were prepared according to the National Research Council (NRC) (1998) standard) and water. Con chickens were immunized according to the normal procedure, and Dis chickens were immunized according to the normal procedure but did not receive the IBV vaccine. At 28 days of age, eye drops and nose drops (the *SX9* strain was administered according to the median embryo lethal dose 10^-5^/0.2 mL) were used for each chicken in the Dis group, and 0.2 mL of sterile saline was used for eye drops and nose drops for each chicken in the Con group. At 1, 5, 11, 18, and 28 dpi, 8 chickens were randomly selected from the Con group and 8 from the Dis group for CO_2_ inhalation euthanasia. Animals that died during the experiment were not used for testing and analysis. In a sterile environment, renal samples were separated and collected quickly. Renal tissue was collected into 2 mL centrifuge tubes and stored at -80°C for RT–qPCR and WB detection. At the same time, renal tissue was collected into 10 mL centrifuge tubes containing 10% formalin for 24 h for histological and pathological examination as well as immunofluorescence analyses (the experimental design is shown in **Figure 1A**).

### Histopathology

The isolated renal tissues were fixed (24 h) with 10% neutral formalin, embedded in paraffin, and sectioned longitudinally and continuously on a microtome with a thickness of 4-5 μm. The samples were stained with hematoxylin and eosin, and the renal structure was observed by microscopy (400 ×).

### Transmission Electron Microscopy

Preparation of specimens for TEM was performed following the method of (Antunez et al. 2011). In brief, the kidney tissue sample was fixed in a 2.5% glutaraldehyde solution, stored at 4°C, and then transferred to an electron microscope solution. Subsequently, dehydration, embedding, sectioning and staining were performed. Finally, the renal structure was observed under a 20,000-fold transmission electron microscope.

### Immunofluorescence Assay

According to the antigen–antibody-specific binding principle, the target protein in kidney tissue was qualitatively located and analyzed. The procedure involves paraffin sectioning, antibody incubation, sealing with glycerin, and then viewing the results under a fluorescence microscope and photographing them. The obtained images were transferred to ImageJ software (National Institute of Health, Bethesda, Maryland, USA). Each microscopic image covered the nucleus (blue) and the positive signal of the target protein (green). The immunofluorescence signal intensity of the target proteins (NF-κB and IFN-γ) was quantitatively analyzed, and the target proteins were locally analyzed. The specific procedures are similar to those described by Amarasinghe et al. (Amarasinghe et al., 2018).

### Real-Time qPCR Analysis

Total RNA was extracted from the kidneys of experimental animals using RNAISO Plus (Takara, Shiga, Japan) reagent strictly following the manufacturer’s instructions. RNA was reverse-transcribed into cDNA using Oligo (DT) reagent (Takara Bio, Hangzhou, China). Primer software (version 3.0; Applied Biosystems, Carlsbad, CA, USA) was used to design fluorescent quantitative PCR primers, and Oligo7 application software was used to evaluate selected primers to select the optimal primer (as shown in **Table 1**). Relative expression was calculated following normalization to GAPDH levels by the comparative delta delta threshold cycle (ΔΔCT) method, and the specific operation steps were performed according to previous research methods (Livak and Schmittgen, 2001).

**Table 1:** Real time -qPCR primers.

Gene	Forward Primer	Reverse Primer
TLR7	F:5’-GCTCCCATCTTGCTCTGGTT-3′	R:5’-ACTTTGGAAACTCACCCAGACT-3′
IRF7	F:5’-CACAAAGCCCAAGGAGTCCA-3′	R:5’-AGTACTCGCAGAACTGGGGA-3′
TRAF6	F:5’-GAAACGGAGACGCTCAGCTA-3′	R:5’-CAGCAACGTCTCCCGTAGAG-3′
IKKβ	F:5’-ATGCAGAAGCTTGCACCAAA-3′	R:5’-CAGCAATGCTCCAGCTGATT-3′
NF-κB P65	F:5’-TTGTGGAGATCCTGGAGCAG-3′	R:5’-AATGGTTTACGCGGATGGTG-3′
IFN-γ	F:5’- ACTGAGCCAGATTGTTTCGAT-3′	R:5’-TCTTTCACCTTCTTCACGCCAT-3′
IL-8	F:5’-GCAAGGTAGGACGCTGGTAA-3′	R:5’-GCGTCAGCTTCACATCTTGA-3′
IL-6	F:5’-AAATCCCTCCTCGCCAATCT-3′	R:5’-CCCTCACGGTCTTCTCCATAAA-3′

### Western Blot Analysis

Protein was extracted from renal tissue using RIPA reagent, and the total protein content was determined by a BCA protein quantitative kit (Solarbio Biotechnology, Beijing, China). SDS–PAGE (TransGen Biotech, Beijing, China) was followed by film transfer and closure and incubation with primary and secondary antibodies; rabbit pAb anti-GAPDH (1:1000, Wanleibio, Shenyang, China), rabbit pAb anti-P65 (1:500, Wanleibio, Shenyang, China), rabbit pAb anti-IFN-γ (1:1000, Wanleibio, Shenyang, China), rabbit pAb anti-IL-6 (1:1000, Wanleibio, Shenyang, China), and rabbit pAb anti-IL-8 (1:1000, Wanleibio), followed by the corresponding HRP-conjugated secondary antibodies (1:5000, Bioss, Beijing, China). Then, the signal was detected with a Bio–Rad Chemidoc Touch imager (Bio–Rad Chemidoc Touch, CA, USA). Finally, the gray value of the corresponding protein was analyzed by ImageJ software (National Institute of Health, Bethesda, Maryland, USA).

### Statistical Analysis

SPSS 23.0 software (SPSS Inc., Chicago, IL, USA) was used for independent-sample t test analysis of all test data. *P*<0.05 indicated a significant difference, *P*<0.01 indicated an extremely significant difference, and *P*>0.05 indicated no significant difference. The results are expressed as the means ± the standard deviation (SD). The number of deaths after NIBV infection was analyzed by the Kaplan–Meier method.

## Results

### Clinical Signs and Pathology

**Figure 2:**
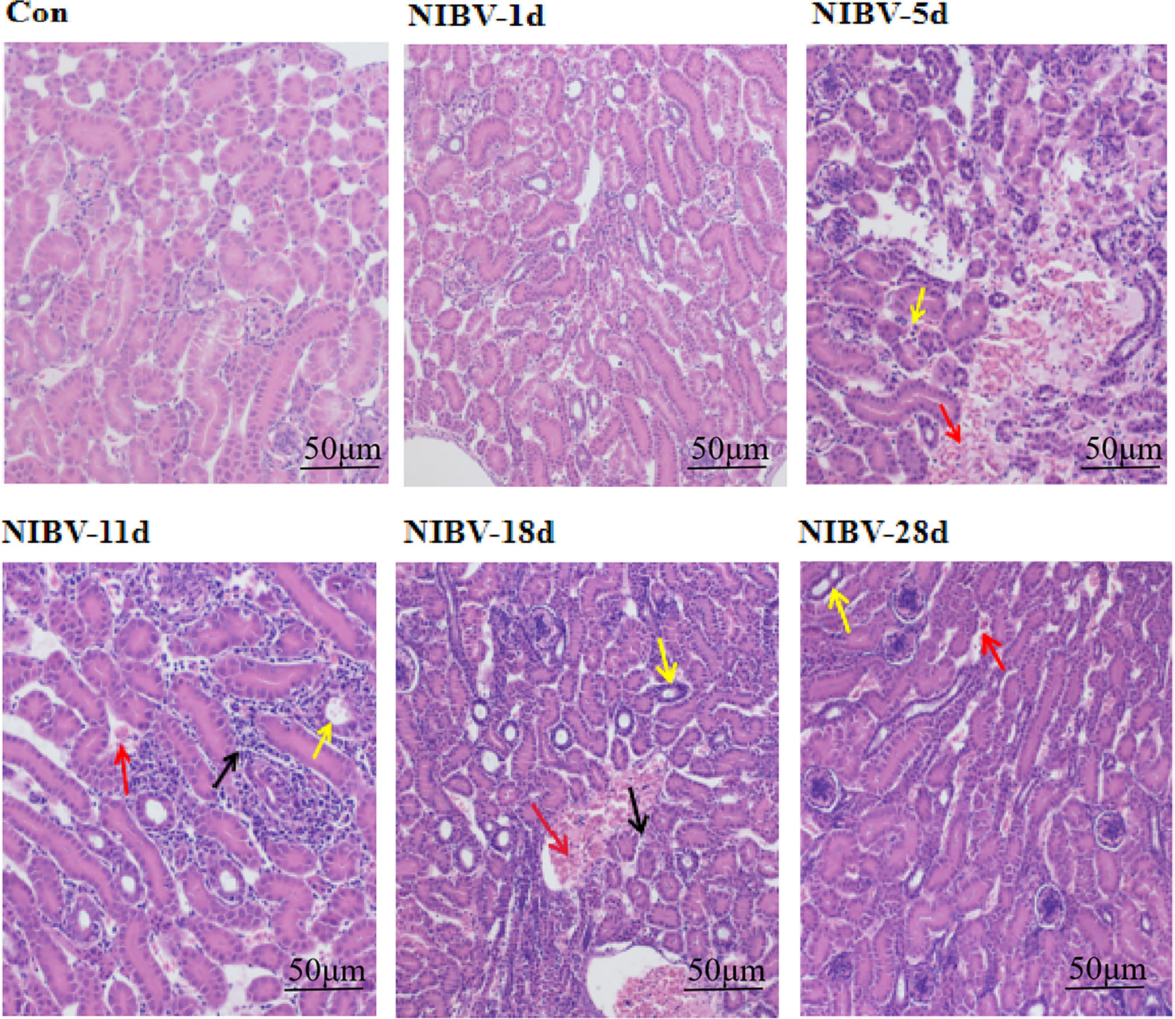
Histopathological changes in the kidneys (H&E staining), the red arrow indicates renal hemorrhage, the black arrows indicate inflammatory cell infiltration; the yellow arrows indicate the absence of tubular structures.

Hy-Line brown laying hens inoculated with the *SX9* strain developed mild clinical signs at 3 dpi, with weakness, cough and ruffled feathers occasionally seen in infected chickens. At 5 dpi, obvious clinical signs appeared, such as cough, diarrhea and drowsiness. Clinical manifestations such as death began at 6 dpi. The Con group did not show any clinical signs (**Table 2**). Analysis of experimental animals infected with NIBV showed an overall mortality rate of approximately 21% (**Figure 1B**); death peaked at 10 dpi, when 20 animals died, with a mortality rate of 15.62%. In dead animals and in some diseased animals, the kidneys were pale and enlarged and had alternated red and white coloring (**Figure 1C**). Microscopic examination of renal sections showed virus particle infection (**Figure 1D**). The virus copy number of kidney tissue increased gradually on the first day after infection to the peak at 11 dpi and then decreased gradually, and the virus content was the lowest at 28 dpi (data not shown in this manuscript). Pathological sections of the renal tissue showed significant renal damage, including loss of tubular structures, bleeding, and significant inflammatory cell infiltration (**Figure 2**). These results indicated that NIBV infection caused kidney injury.

**Table 2:** Clinical symptoms of control and diseased chickens infected with NIBV SX9.

The infection of time	Con group	Dis group
1 dpi	normal	no apparent clinical signs
3 dpi	normal	weak, cough, ruffled feather
5-6 dpi	normal	Coughing, diarrhea, drowsiness, and death
8-11 dpi	normal	moderately depressed, incoordination, peak of death
14-16 dpi	normal	Chickens start to recover (A gradual improvement in diet)
18-28 dpi	normal	The surviving chickens returned to normal

### TLR7/NF-κB Signaling Axis mRNA Expression Responded to NIBV Infection in Renal Tissue

**Figure 3:**
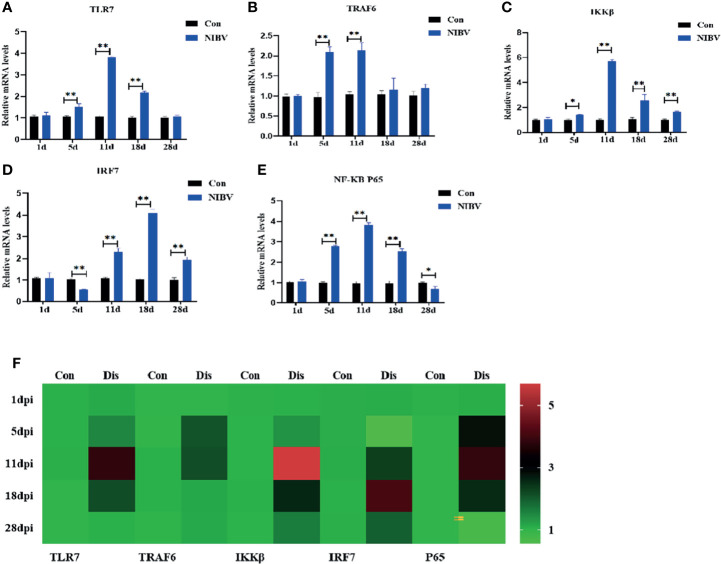
Effects of NIBV on expression levels of TLR7/NF-κB signal axis related factors in chicken kidney tissue. **(A–E)** TLR7, TRAF6,IKKβ,IRF7 and NF-κB P65 mRNA expression levels; **(F)** Heatmap analysis of time and TLR7-related genes mRNA expression levels. *(0.01 < P < 0.05), **(P < 0.01), (N=6).

The changes in TLR7/NF-κB signaling pathway-related genes in renal tissue after infection with the *SX9* strain are shown in **Figure 3**. Compared with those in the Con group, the mRNA expression levels of TLR7 were upregulated at 5 to 18 dpi, and the mRNA expression levels of TRAF6 were upregulated at 5 dpi and 11 dpi, with no significant changes at the remaining time points (**Figures 3A, B**). After NIBV infection, the mRNA expression levels of IRF7 and IKKβ did not change significantly at 1 dpi but were significantly upregulated at 11 to 28 dpi (**Figures 3C, D**). For the mRNA expression levels of NF-κB P65, there were no significant changes at 1 dpi, which increased significantly at 5 to 18 dpi and decreased significantly at 28 dpi (**Figure 3E**).

### NIBV Infection Altered TLR7/NF-κB Signaling Axis-Related Protein Expression

**Figure 4:**
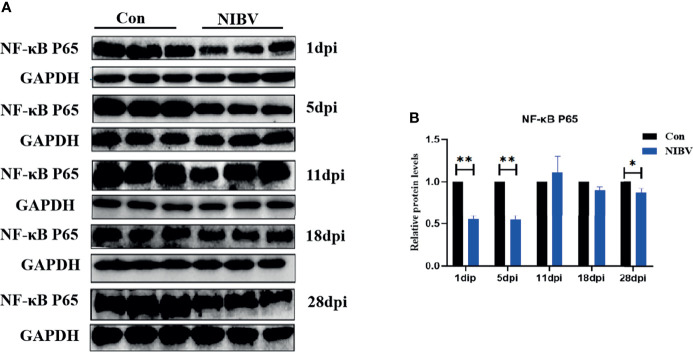
**(A)** NF-κB P65 Protein band graph; **(B)** NF-κB P65 protein expression levels. *(0.01 < P < 0.05), **(P < 0.01), (N=3).

**Figure 5:**
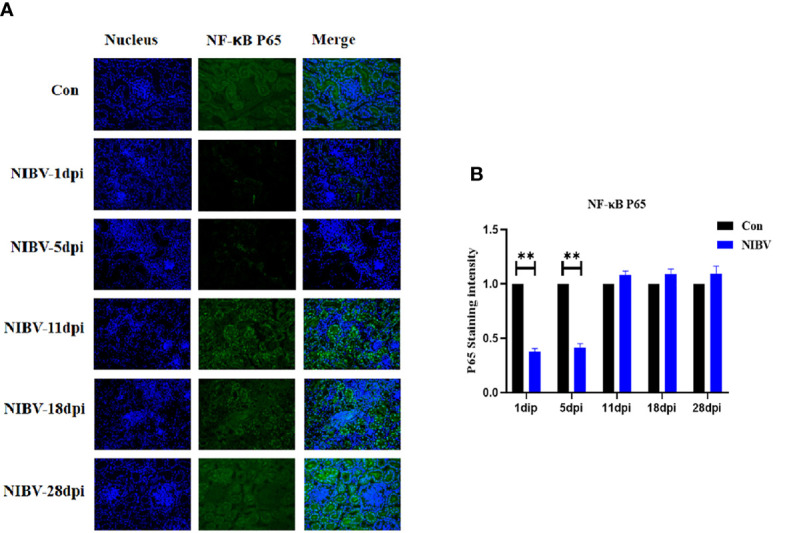
Qualitative and quantitative immunofluorescence analysis of NF-κB P65 proteins. **(A)** In this image, the nucleus staining is shown in blue, the NF-vB P65 stained is shown in green, and the combined images show the co-localization signal (400 total magnification). **(B)** Mean NF-κB P65 staining intensity in each group. Data are expressed as the mean SD, *(0.01 < P < 0.05), **(P < 0.01), (N=3).

Western blotting results showed that the expression of NF-κB P65 protein in kidney tissue was decreased overall after *SX9* strain infection, except at 11 dpi, and at 1 and 5 dpi, it was significantly lower than that in the normal group (**Figure 4**). We also investigated the localization change of NF-κB P65 after NIBV infection. FITC fluorescence was used to visualize NF-κB P65 (green), with nuclear staining shown in blue (**Figure 5**). There was less NF-κB P65 signal at 1 dpi and 5 dpi than in the Con group, and the protein was mainly localized in the cytoplasm. At the later stage, the NF-κB P65 signal was significantly increased compared with that at the early stage, and green fluorescence was predominantly located in the nuclei (**Figure 5**). These results proved that NIBV infection alters P65 protein expression and translocation to the nucleus, which in turn may promote downstream gene expression.

### Expression of Innate Immune Cytokine mRNAs in Renal Tissues After NIBV Infection

**Figure 6:**
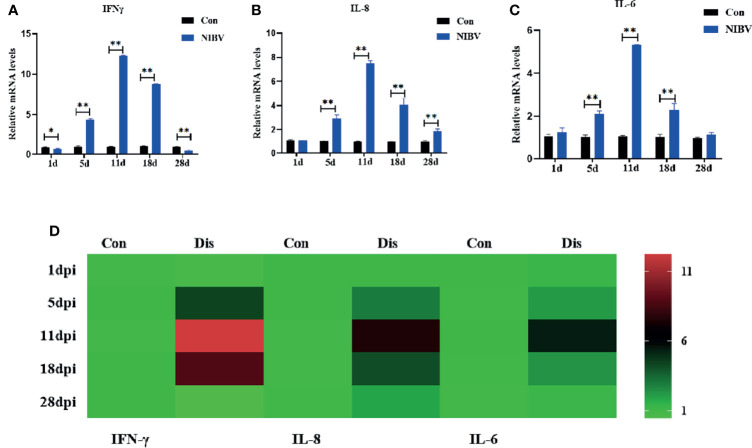
Effects of NIBV on the expression of inflammatory factors in chicken kidney tissue. **(A–C)** IFN-γ, IL-8 and IL-6 mRNA expression levels; **(D)** Heatmap analysis of time and innate immune-related genes mRNA expression levels. *(0.01 < P < 0.05), **(P < 0.01), (N=6).

The expression profiles of innate immune cytokines in renal tissue after infection with the *SX9* strain are shown in **Figure 6**. Compared with those in the Con group, IFN-γ mRNA levels were significantly downregulated at 1 dpi and 28 dpi but reversed at 5 to 18 dpi (**Figure 6A**). The expression of IL-8 mRNA showed the same trend as those of IKKβ, with no significant change at 1 dpi and significant increases at 5 to 28 dpi (**Figure 6B**). The expression of IL-6 mRNA was consistent with that of IL-8 mRNA from days 1 to 18 after infection, and there was no significant change at 1 dpi but a significant increase at 5 to 18 dpi (**Figure 6C**).

### Effects of NIBV Infection on the Protein Levels of Innate Immune Cytokines

**Figure 7:**
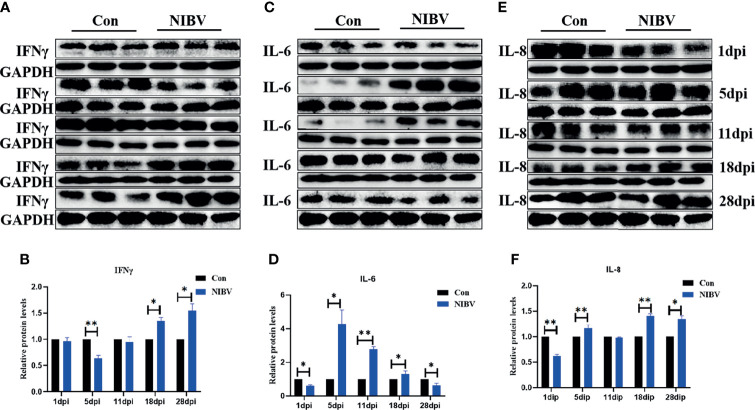
**(A, C, E)** Protein band graph; **(B, D, F)** IFN-γ, IL-6 and IL-8 protein expression levels. *(0.01 < P < 0.05), **(P < 0.01), (N=3).

To verify the role of NIBV infection on innate immune cytokines, the protein levels of IFN-γ, IL-6 and IL-8 were detected by western blotting (**Figure 7**). IFN-γ protein expression at 5 dpi was significantly lower than that in the Con group, then gradually increased and remained increased at 28 dpi. IL-6 protein was significantly lower than that in the Con group at 1 dpi, significantly elevated at 5 to 18 dpi, and decreased significantly at 28 dpi. The expression of IL-8 protein was significantly lower than that of the Con group at 1 dpi, increased again at 5 dpi, 18 dpi and 28 dpi, and was significantly higher than that of the normal group in the later stage of infection.

**Figure 8:**
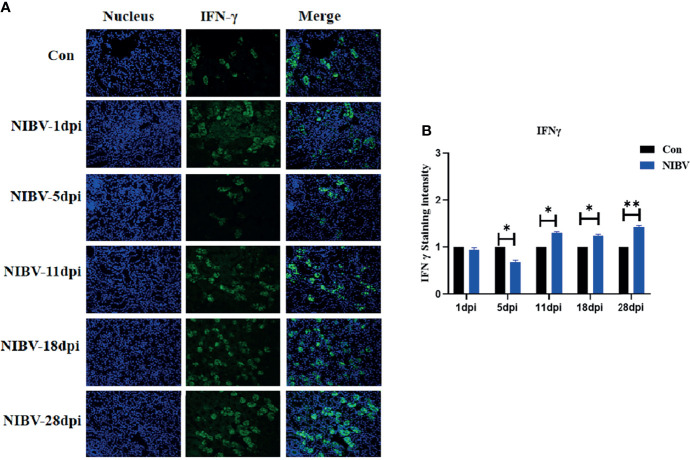
Qualitative and quantitative immunofluorescence analysis of IFN-γ proteins. **(A)** In this image, the nucleus staining is shown in blue, IFN-γ staining is shown in green, and combined images show co-localization signal (400 total magnification); **(B)** Mean IFN-γ staining intensity in each group. Data are expressed as the mean SD, *(0.01 < P< 0.05), **(P < 0.01), (N=3).

To investigate the change in natural immune factors after NIBV infection, immunofluorescence was used to analyze IFN-γ in the infected kidney tissues (IFN-γ is green; nucleus is blue) (**Figure 8A**). IFN-γ positive signals were mainly distributed in the nucleus. The positive signal intensity of IFN-γ had no significant change at 1 dpi and decreased significantly at 5 dpi; after that, the positive signal of IFN-γ increased gradually, from 11 dpi to 28 dpi, which was significantly stronger than that in the normal group (**Figure 8B**).

## Discussion

The kidney is the main target organ for NIBV infection, and the outbreak of COVID-19 in the past two years can also directly induce kidney disease (Santoriello et al., 2020), which further highlights the need for research on the relationship between coronavirus and innate immunity. There is also increasing evidence that the TLR7/NF-κB signaling pathway plays an important role in innate immunity, but the relationship between NIBV and the TLR7/NF-κB pathway and its precise regulatory mechanism remain unclear. In this study, we reported the results of renal tissue section observation, ultrastructural observation, immunofluorescence and related gene proteins in chickens infected with NIBV. The results suggest that NIBV activates the TLR7/NF-κB signaling axis in renal tissue, thereby promoting cytokine production. This may provide important information for future studies on how coronavirus infection induces innate immune responses.

We observed the renal tissue structure under a microscope, which showed that there were multiple coronavirus particles in the renal tissue, and the renal tubules were missing, with a large number of infiltrating inflammatory cells. Autopsy examination showed red and white spotted kidneys at 11 dpi, and these results are consistent with previous studies (Lin et al., 2015; Xu et al., 2019). This indicates that we successfully established the model of coronavirus NIBV. However, the mechanism by which NIBV mediates renal injury through the TLR7 signaling pathway is still unclear, so we detected the TLR7 signaling pathway according to the pathogenic characteristics of NIBV. The innate immune system emerged in the early stages of evolution, and Toll-like receptors play a key role in innate immunity. TLR7 is a single-stranded RNA (ssRNA) sensor in innate immunity and is one of the most important pattern recognition receptors (Mukherjee et al., 2016 , Zhang et al., 2016). The experimental results of this study showed that after NIBV infection, TLR7 mRNA levels were significantly upregulated at multiple time points, consistent with the degree of kidney injury observed at different time points. It has been suggested that NIBV infection can promote TLR7 mRNA expression, induce the immune response and mediate kidney injury in the body, which is consistent with the idea proposed by He et al. (He et al., 2016a) that the TLR7 receptor is activated after M41 IBV infection. However, in this study, the expression of TLR7 mRNA was somewhat different from that of M41 IBV in the later stage of infection, possibly due to the difference in the type of virus. Studies have shown that different types of IBV infection can induce different expression levels of inflammation-related genes (Jang et al., 2013; Okino et al., 2017), indicating that different pathogenic types of IBV infect hosts with different innate immune responses in different target organs. These results suggest that NIBV infection promotes TLR7 mRNA expression.

Tumor necrosis factor receptor associated factor 6 (TRAF6), a conjugated protein that mediates NF-κB activation, has been identified as an immunomodulatory actor downstream of the Toll receptor family (Walsh et al., 2015; Brenke et al., 2018). In addition, IKKβ is considered to be an essential regulator of NF-κB, and IKKβ forms a dimer with IKKα and plays a leading role in the regulation of NF-κB activity through protein phosphorylation (Page et al., 2017). IRF7 and NF-κB have similar functions; an important regulator of the innate immune response is activated by phosphorylation after viral infection and translocates into the nucleus after homologous dimerization or allodimerization to initiate transcription expression of inflammatory factors (Yu et al., 2018). Overall, these genes are downstream factors of the TLR7 gene and promote the expression of inflammatory cytokines. We found that the downstream genes TRAF6 and IKKβ were significantly upregulated in renal tissues throughout the experiment, and the expression trend of TLR7 was the same; however, the increase in TRAF6 was not obvious in the later stage, suggesting that SX9 inhibited the expression of TRAF6 in the later stage of kidney infection. Studies have shown that TRAF3 and TRAF6 complexes are inactivated by pathogenic microorganisms to limit or terminate proinflammatory cytokines and interferons and to evade host innate immunity (Panda et al., 2015). The mRNA level of NF-κB P65 was consistent with the mRNA expression trends of the upstream genes TRAF6 and IKKβ. At 28 dpi, however, the NF-κB P65 mRNA level was the opposite, which may be mainly dependent on the IRF7 nuclear transcription factor at the late stage or NF-κB P65 adapted to the host at the late stage. These findings were also consistent with the results of IRF7 in this experiment, which showed a significant increase in the level of IRF7 mRNA at 28 dpi. The NF-κB P65 protein level was opposite to the NF-κB P65 transcription level and decreased after NIBV infection. It may be that coronavirus infection inhibits the production of host antiviral proteins but does not affect mRNA expression (Huang et al., 2011). The fluorescence intensity of NF-κB p65 at 1 dpi and 5 dpi was lower than that of the Con group, and the target protein was mainly located in the cytoplasm. The intensity of the NF-κB P65 fluorescence signal was stronger in the middle and late stages than in the early stage, and the target protein was mainly distributed in the nucleus. These results proved that NIBV infection alters P65 gene expression and translocation to the nucleus. It was further demonstrated that NIBV infection promoted TLR7 mRNA expression, activated the downstream factors TRAF6 and IKKβ, and then activated nuclear transcription factors.

IFN-γ, as the only type II interferon that has been characterized, is a key regulatory protein for the function of the entire immune system (Wheelock, 1965; Fenimore and A, 2016). IFN-γ is mainly secreted by NK cells, is a typical representative proinflammatory factor, and can also promote the secretion of other cytokines (IL-6, IL-8) (den Hartog et al., 2020). In summary, IFN-γ, IL-8 and IL-6 are innate immune cytokines that play an important role in the body’s natural immunity. The results of this study are consistent with those of other viral infections (Pananghat et al., 2016; Li et al., 2020). IL-6 and IL-8 mRNA expression levels were significantly upregulated (consistent with the observation of H&E staining, which increased first and then decreased). In the early stage, the IL-8 and IL-6 protein levels were different from the transcription levels, mainly because the mRNA expression in the renal tissue was not upregulated after NIBV infection, so the protein expression was significantly decreased in the early stage of infection and was even significantly lower than that in the Con group. At 11 dpi, IL-8 protein levels were inhibited by high viral levels. It has also been reported that the IBV S protein can interact with the initiation factor EIF-3F, leading to the inhibition of general protein synthesis (Kint et al., 2016). These results suggest that NIBV infection activates IL-8 and IL-6. The expression of IFN-γ mRNA was inhibited at first and then significantly increased under NIBV infection and then significantly decreased at 28 dpi. In the early stage, this change may be due to the stress response caused by challenge and fasting before sampling, and in the later stage, it is mainly because the host has adapted to the environment *in vivo* and *in vitro*. The IFN-γ protein level was generally consistent with mRNA expression but with obvious hysteresis. In this study, the expression levels of the cytoinflammatory factors IFN-γ, IL-8, and IL-6 were consistent with the trends of the nuclear transcription factors (NF-κB P65 and IRF7). Moreover, we found consistent responses to changes in the TLR7/NF-κB signaling axis, innate immune cytokines and histopathology throughout the course of infection. These results suggest that NIBV infection activates the TLR7 signaling axis and further activates the downstream pathway NF-κB, thereby inducing the production of inflammatory cytokines and chemokines.

## Conclusion

**Figure 9:**
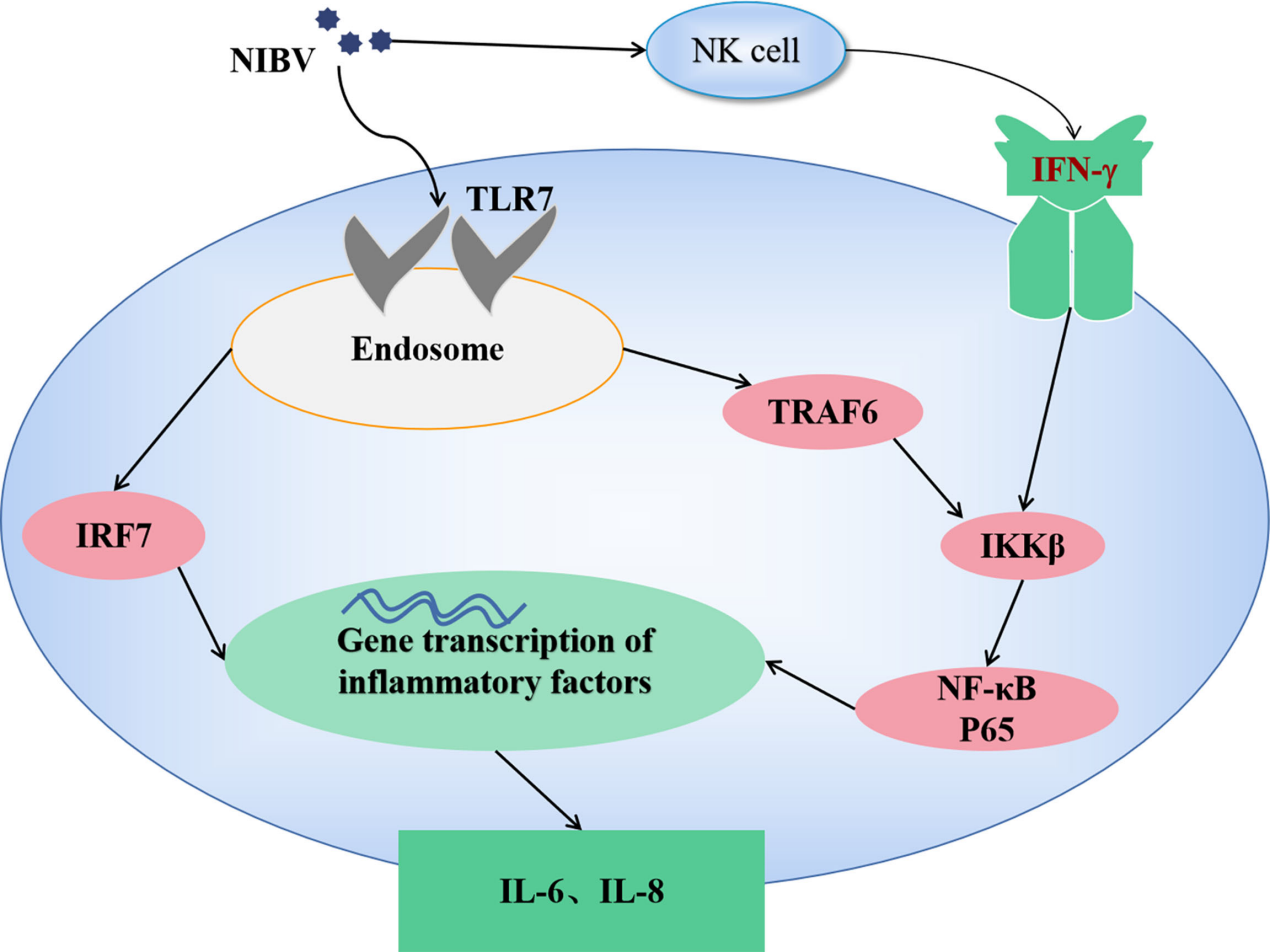
The mechanism of TLR7/NF-κB signaling axis in NIBV infection.

In conclusion, infection with the NIBV SX9 strain activates the TLR7 signaling axis in target tissue, activates the nuclear transcription factors IRF7 and NF-κB, induces the production of proinflammatory cytokines, causes an inflammatory response and leads to kidney damage. Our present results suggest that the natural immune response plays an important antiviral role in NIBV infection. According to this study, a signal diagram of TLR7/NF-κB was drawn (as shown in **Figure 9**).

## Data Availability Statement

The original contributions presented in the study are included in the article/supplementary material. Further inquiries can be directed to the corresponding authors.

## Ethics Statement

The Institutional Animal Care and Use Committee of Jiangxi Agricultural University approved these animal experiments, and all animal experiments adhered rigorously to the animal care guidelines of Jiangxi Agricultural University (approval ID: JXAULL-202112; approval date: 6 August 2021). All the birds were put down using carbon dioxide euthanasia, and all attempts were made to minimize the suffering of the animals.

## Author Contributions

NL: Methodology; Writing-original draft; Writing-review and editing. CH: Methodology; Visualization; Writing-review and editing. XNG: Conceptualization; Methodology; Visualization; Writing-review and editing. XQG: Funding acquisition; Writing-original draft; Writing-review and editing. All authors contributed to the article and approved the submitted version.

## Conflict of Interest

The authors declare that the research was conducted in the absence of any commercial or financial relationships that could be construed as a potential conflict of interest.

## Publisher’s Note

All claims expressed in this article are solely those of the authors and do not necessarily represent those of their affiliated organizations, or those of the publisher, the editors and the reviewers. Any product that may be evaluated in this article, or claim that may be made by its manufacturer, is not guaranteed or endorsed by the publisher.
